# Exploring the roles and functions of champions within community-based interventions to support older adults with chronic conditions: A scoping review protocol

**DOI:** 10.1371/journal.pone.0291252

**Published:** 2023-10-13

**Authors:** Kristina M. Kokorelias, Hardeep K. Singh, Reham Abdelhalim, Marianne Saragosa, Guillaume Lim Fat, Christine Sheppard

**Affiliations:** 1 Division of Geriatric Medicine, Department of Medicine, Sinai Health System and University Health Network, Toronto, Ontario, Canada; 2 Department of Occupational Science & Occupational Therapy, Temerty Faculty of Medicine, University of Toronto, Toronto, Ontario, Canada; 3 National Institute on Ageing, Toronto Metropolitan University, Toronto, Ontario, Canada; 4 Rehabilitation Sciences Institute, Temerty Faculty of Medicine, University of Toronto, Toronto, Canada; 5 KITE Toronto Rehabilitation Institute-University Health Network, Toronto, Ontario, Canada; 6 Joseph Brant Hospital, Burlington, Canada; 7 Burlington OHT, Burlington, ON, Canada; 8 Lunenfeld-Tanenbaum Research Institute, Sinai Health, Toronto, Ontario, Canada; 9 Factor-Inwentash Faculty of Social Work, University of Toronto, Toronto, Canada; University of Kwazulu-Natal, SOUTH AFRICA

## Abstract

**Background:**

Health care solutions are needed to meet the need of an ageing population. Health care champions are people who endorse the adoption of new initiatives being implemented within health care settings. Although the role of champions has been cited as key to the success of numerous community-based interventions implemented to improve the care of older adults with chronic conditions, no synthesis of their implementation experiences have been conducted. We report on a scoping review protocol that will be applied to collect evidence on the role of champions within community-based health interventions to support older adults with chronic conditions. Specifically, we will identify how the term ‘champion’ is used and defined (i.e., conceptualized) and identify the roles (i.e., professional background) and functions of champions (i.e., responsibilities). We will also explore how this role impacts program implementation.

**Methods:**

This is a scoping review protocol informed by guidelines for Scoping Reviews (PRISMA-ScR) and a six-stage scoping review methodology. Peer-review literature will be retrieved from Medline, CINAHL, PubMed, PsycInfo, Cochrane JBI and Scopus databases, using a peer-reviewed search strategy developed in collaboration with an Information Specialist. The scoping review will consider all empirical studies published in English. Two reviewers will pilot-test the screening criteria and data abstraction forms, and then independently screen the literature. Extracted data will be analyzed numerically and thematically. Self-identified champions will be consulted to refine the practice recommendations from this work.

**Discussion:**

This scoping review will broadly and systematically identify, define and expand existing knowledge on champions’ impact in implementing community-based interventions to support older adults with chronic conditions. We anticipate that our results will lead to a greater understanding of the characteristics and role champions play within these interventions, which will be relevant to a wide range of knowledge users, including researchers, decision-makers, and health care providers.

## Introduction

Global life expectancy has increased substantially over the past decades, resulting in more older adults (defined as persons *≥* 65 years old*)* living in developed and developing countries [1]. An increase in global life expectancy and population ageing is accompanied by a growing proportion of older individuals living with chronic conditions [2]. Caring for many older adults with multimorbidity impacts the healthcare systems of many countries [3]. Whereas health care has historically relied heavily on the acute care model, an increase in chronic illnesses necessitates novel models of care and research that prioritize prevention and overall wellbeing over illness trajectories [4, 5]. Moreover, many older adults live with several chronic illnesses (i.e., multi-morbidity [6]; e.g., a combination of heart failure, vascular disease, stroke, diabetes, hypertension, chronic obstructive pulmonary disease, renal disease, and dementia) [7] that are confounded by psycho-social needs, such as mental health or social isolation [8, 9]. Consequently, there is growing evidence that community-based chronic disease management programs can help older adults improve their health outcomes while reducing healthcare utilization [10, 11].

In an attempt to contain the rising healthcare costs associated with the care of older adults, many health care systems are investing in community-based services [12–15]. Community-based care often aligns with the preferences of older adults, who desire to age in their homes and communities [16]. Numerous community-based services exist for older adults with chronic conditions (e.g., [17–21]). Community-based interventions for older adults with chronic illness often aim to improve symptom and medication management [22] and mental health and wellbeing [23–25] or reduce health care utilization [10, 25], while improving access to appropriate and timely care [26, 27]. The health services provided in the community range from targetted self-management or general health interventions including remote care monitoring [28, 29] to physical activity, nutrition and active social participation interventions [21, 30, 31]. In addition, community-based interventions targeting chronic disease management provide specialized home-care services, including medication delivery, assistance with instrumental activities of daily living and complex patient care tasks [2, 32, 33]. Community-based health interventions are provided by a broad range of health and social care providers, such as nurses, physicians, social workers, pharmacists, and personal support workers [34].

Studies focusing specifically on implementing community-based interventions for older adults with chronic conditions are rare but growing [18, 35, 36]. Existing reviews have explored the implementation of community-based interventions for older adults with chronic conditions and noted the important role of stakeholders in facilitating the implementation [18, 37]. Some of these stakeholders may be known as champions [38]. Champions have been defined within the field of implementation science as “individual(s) who dedicate themselves to supporting, marketing, and driving through an implementation, overcoming indifference or resistance that the intervention may provoke in an organization” [39] (pg.9). Champions are thought to be key to the implementation of numerous interventions for older adults with chronic conditions [40–42] and their contributions to health service interventions are beginning to be recognized [43–45]. Champions have been posited as being inturmental in intervention implementation,by providing support to other members in their community so that they can access the intervention [46] and faciliating partner engagement to support intervention implementation and deliver the intervention [47]. Thus, champions have been reported to influence the uptake and success of community-based interventions [48, 49]. However, the term "champion" is also used in many ways by those involved in health service intervention development and implementation.

Although champions are believed to be valuable in implementing community-based interventions for older adults with chronic conditions, it is unclear what their role entails or how their role contributes to implementation success [50]. While research on champions is emerging in some healthcare contexts (e.g., [42, 50–53]), there remains a lack of understanding of the unique role of champions within the context of community-based interventions for older adults. In the context of hospital-based interventions, champions support the communication and persuasion of the intervention, provide accountability for intervention success, motivate people to partake in the intervention and encourage learning about the intervention [50, 54]. The literature from other healthcare contexts may not be applied to the unique needs of community-dwelling older adults [55], nor account for unique policies and procedures within community-based health care interventions [56, 57]. Examining how champions have supported community-based interventions can inform future intervention development [42]. As such, the overarching aim of our study is to synthesize existing literature on role and functions of champions within novel community-based interventions to support older adults with chronic conditions. Specifically, we will identify: i) how the term ‘champion’ is used and defined (i.e., conceptualized) within this literature, ii) the roles (i.e., professional background) and functions of champions (i.e., responsibilities) and iii) how champions impact on program implementation (i.e., reported links between the champion and program outcomes and any barriers to integrating champions within interventions effectively).

## Methods

### Design

We will conduct a scoping review of peer-reviewed literature informed by the framework outlined by Arksey and O’Malley [58] and refined by Levac et al. [59]. A scoping review was selected due to the heterogeneous nature of the research questions [58], Moreover, scoping reviews are appropriate when the research question is broad and multifaceted [58]. This framework will guide the research team to perform the scoping review in six-stages: (1) identifying the research question; (2) identifying the relevant studies; (3) study selection; (4) charting the data; (5) collating, summarizing, and reporting the results; and (6) consultation. The Scoping review extension of the Preferred Reporting Items for Systematic Reviews and Meta-Analyses (PRISMA-Scr) also helped to guide the conduct of the review and will be used to report the results [60].

### Research question

We will examine the following research question: What are the extent, range, and nature of the evidence available in peer-reviewed literature on the roles, functions and value of champions in implementing community-based interventions for older adults with chronic conditions? The following sub-questions will also be explored: (1) how the term ‘champion’ is used (i.e., operationalized) and defined (i.e., conceptualized) within this literature?; (2) what are the roles (i.e., professional background) and functions of champions (i.e., responsibilities) of community-based interventions for older adults with chronic conditions?; and (3) how do the roles of champions impact program implementation the implementation of community-based interventions for older adults with chronic conditions? Older adults will be broadly defined as people aged 65 years or older. Community-based health interventions will be defined as health interventions offered in community settings, other than in institutional settings, such as hospitals, nursing homes, and assisted living facilities, that also require health care providers to have a role in the intervention [61]. We will include community-based health interventions that occur in primary care (e.g., community health centres) [62]and/or through virtual modalities [63].

### Search strategy

We will search for English language peer-reviewed journal articles published from all countries between January 2013 and the present day. The limited date range was selected as we are interested in recent approaches to implementation, such that this information can be most relevant for future community-based interventions for older adults with chronic conditions. Databases to be searched for the study will include: Web of Science, Scopus, JBI, PsychInfo, PubMed (non-Medline), Cochrane CINAHL, CINAHL Complete, Medline, and Medline Complete. We will follow the recommendations made by Levac et al. [59] to conduct an iterative and team-approach to refine the search strategy. The search strategy will be co-developed between the research team and an Information Specialist using three keyword groups to capture the related terms for the search as it relates to the topic (Champion), setting (Community) and age of the population (geriatrics). The Information Specialist will develop a search string using an “AND” Boolean operation for each database based on these terms. An initial attempt at a search strategy was conducted on March 3, 2023 in Medline and resulted in 1296 results. This initial search demonstrated that the key terms selected were likely to identify results related to the focus of this scoping review (**see S1 Appendix**). The Information Specialist will complete all database searches, who will be supervised and guided by two authors (KMK, CLS). However, to ensure that the scoping review captures a comprehensive breadth of literature, the research team will also extensively hand-search various reference lists of included studies and recent issues of key journals (e.g., the Implementation Science Journal, Implementation Science, Implementation Science Communications and BMC Health Services Research, Implementation Research and Practice) [64].

### Study selection

All reviewers will screen the first 30–50 titles/abstracts and 5–10 full papers for calibration and consistency. At this stage, any modifications or clarity to the inclusion criteria will be made [59]. Next, the review process consists of two stages. The first stage will screen titles and abstracts and the second stage will consist of full-text screening. Two research assistants and the first author (KMK) will review the articles (i.e., double-review) [65] and will be supervised closely by another author (CLS). The research assistant will review the selections under review with ‘yes,’ ‘no’ or ‘maybe’ using Covidence software [66]. To reduce the risk of missing eligible studies, a third reviewer will check those excluded at the full paper screening stage (KMK or CLS). Disagreements and uncertainties will be discussed with a third investigator (CLS). If a resolution cannot be researched, we will discuss the conflicts with the entire research team to resolve them.

As some community interventions may not be specifically targeted towards older adults, despite them being the population that derive the most benefit from them due to frailty, comorbidity, and pscychosocial vulnerabilities, we are choosing to review all included community interventions. The following inclusion criteria will be used to guide the search and article selection: (1) report the implementation process or evaluation of a community-based intervention (e.g., intervention in the community) aimed at supporting adults with chronic conditions or multimorbidity, with at least 50% of the targeted population being older adults; (2) uses the term ‘champion’ within the title or body of the manuscript; (3) published empirical studies that use quantitative, qualitative or mixed methods; (4) written in English; and (5) from 2013 onwards. Exclusion criteria are: (1) literature reviews or conference abstracts; (2) articles without the description of the role of the champion or commentaries of interventions not yet implemented; and (3) empirical studies that focus on the impact or implementation of a community-based intervention for older adults with chronic conditions, but do not examine the involvement of champions in the implementation process. Any potentially eligible studies with insufficient information to include in the review will also be excluded.

### Charting the data

Data from the included articles will be charted and sorted according to key variables related to the research question, including: author, title, study methodology, the country where the study was conducted, the research aims, methods, sample size, chronic condition of focused, champion definitions or conceptualizations, a description of the implication, study setting (e.g., any institution or community setting), the role and functions of the champion, indicators of champion’s impact, other facilitators to implementation and/or the implementation approach (if specified) and key findings of the study. Key findings of the studies will specifically report on the study’s results on the champion’s involvement and outcomes of that involvement in program implementation. As consistent with scoping reviews, the quality of the included studies will not be assessed [67]. As charting is an iterative process, the final charting form will be developed in consultation with the entire research team, who will have greater clarity about key variables as the review process progresses [59]. Charting will be conducted by one of two research assistants and reviewed by the lead researcher (KMK). We will chart extracted data using a customized data charting form on Covidence software [66].

### Collating, summarizing and reporting the results

Our research team will follow these steps posed by Levac et al [59] to help collate, summarize and report the results: (1) analyse extracted data using a numerical summary analysis and qualitative thematic analysis; (2) disseminate the results of the scoping review; and (3) discuss implications for future research, policy and practice. Content analysis will support the integration of qualitative and quantitative studies [68]. Firstly, the charted data will be reviewed by the entire research team and initial ideas for codes will be recorded and shared at a team meeting. Next, the preliminary thoughts will be consolidated into a codebook with deemed important examples [68, 69]. Thirdly, these codes will be applied to all articles through a coding process managed by NVivo software [70] and facilitated by a research assistant. Once all data is coded, the research team will host a series of meetings with the goal to organize the coded information into categories relevant to the review’s research questions. Categories will be reviewed and refined, until final categories, with agreement from the entire research team, are established, defined and named. The results will be disseminated through a peer-reviewed journal and implications for future research, policy and practice will be presented at local, national and international conferences on aging. All research team members have established relationships with various community-based healthcare agencies, which we will also use to disseminate findings.

### Consultation

Following recommendations for scoping review methodology [58, 59], we will host a consultation meeting via an online video-conferencing platform with self-identified champions of existing community-based health interventions for older adults with chronic conditions. Consultation with exiting champions will involve sharing study aims and working drafts of the recommendations from the literature review with stakeholders for feedback. To the best of our ability, we will seek variability in the consultation participants, by roles (e.g., executive vs front-line workers) Data from the consultation activity will be incorporated into the review’s final recommendations. The purpose of the consultation is to support the interpretation of the literature further and help support the overarching aim of developing recommendations on how to use champions more effectively in the context of community-based interventions to support older adults with chronic conditions.

## Limitations

Our scoping review protocol has a few noteworthy limitations. First, we have limited the included studies to those disseminated in English, and published within the last decade. As such, there is an increased risk of missing relevant articles reported in languages other than English. In addition, our results may not be generalizable to other contexts as we will likely have a bias toward studies from English-speaking and high-income countries. Community-based interventions, especially in low-resource settings, play a crucial role in addressing public health challenges and improving health outcomes [71, 72]. We acknowledge that this language bias may result in the omission of important research from low- and/or middle-income countries and other low-resource settings, where community-based interventions are highly relevant and needed Moreover, this study will not consider the quality of articles, potentially limiting the scope of recommendations being made for policy or practice. Nonetheless, we will generate recommendations based on a comprehensive sample of peer-reviewed evidence [73]. We will be mindful to interpret our findings with caution, emphasizing the need for context-specific adaptations of community-based interventions and considering the unique challenges and resources available in low- and/or middle-income countries and other low-resource settings.

## Discussion

Champions have been called “special people” by scholars (pg.240) who believe them to be the key success factor behind the implementation of various new services being offered in health care settings [74]. Champions are so widely promoted in health care interventions as a key to implementation success. Thus, understanding who these individuals are, their roles and the key functions of their roles that lead to successfully implemented interventions within the growing context of community-based interventions to support older adults with chronic conditions is needed. This study will be the first step to developing a comprehensive understanding of the roles involved as champions and their experiences during implementing community-based interventions to support older adults with chronic conditions. Understanding the factors that lead to successful implementation, as facilitated by champions, can enhance the effectiveness and sustainability of these interventions. Thus, this information can guide the planning of implementation processes for future community-based interventions to support older adults with chronic conditions. This exploratory study will also highlight avenues of further investigation into the role of champions in implementing community-based interventions to support older adults with chronic conditions. For example, this knowledge can inform the development of targeted training and support programs for champions, ultimately enhancing their capacity to lead and drive successful intervention implementation [48]. By recognizing the pivotal role of champions, healthcare organizations, policymakers, and practitioners can prioritize the cultivation and support of these individuals, maximizing the impact of community-based interventions and improving the overall well-being and care of older adults [47, 48].This study and the subsequent future research can offer new insight into champions’ roles and functions and their key importance within the design and implementation of community-based interventions to support older adults.

## Supporting information

S1 File (DOCX)

S1 Appendix Ovid Medline(R) all. (DOCX)
