# Supplementary material for: Exploring the roles and functions of champions within community-based interventions to support older adults with chronic conditions: A scoping review protocol
Source: PLoS One. 2023 Oct 13;18(10):e0291252. doi: 10.1371/journal.pone.0291252 (PMC10575514; doi:10.1371/journal.pone.0291252)
Supplement: S1 File — (DOCX) [file pone.0291252.s001.docx]

# Search for: Exploring the roles and functions of champions within community-based interventions to support older adults with chronic conditions: a scoping review

# All databases searched and exported on May 31, 2023.

| **Databases** | **Number of Results** |
| --- | --- |
| Ovid Medline ® All | 2025 |
| Ovid’s APA PsycINFO | 932 |
| Ovid’s JBI EBP | 145 |
| EBSCO’s CINAHL Complete | 4490 |
| Wiley’s Cochrane Library | 574 |
| NLM’s PubMed (Non-Medline) | 466 |
| Elsevier’s Scopus | 3958 |
| Clarivate’s Web of Science Core Collection: Sci-Expanded, SSCI, AHCI, CPCI-S, CPCI-SSH, BKCI-S, BKCI-SSH, ESCI | 1454 |
| **Total Before Upload to Covidence** | 14044 |
| Total After Deduplication in Covidence |  |
| Duplicates |  |

## **OVID Medline:**

**Results Filtered to May 2013 onwards**

Ovid MEDLINE(R) ALL <1946 to May 30, 2023>

1 community health services/ 33074

2 community mental health services/ or community pharmacy services/ 24767

3 hospitals, community/ or hospitals, public/ 29322

4 *Delivery of Health Care/ 67083

5 ((communit* adj2 care) or (communit* adj2 intervention*) or (communit* adj2 treatment*) or (communit* adj2 health) or (communit* adj3 service*) or (communit* adj2 mental health*) or (communit* adj2 pharmacy) or (communit* adj2 pharmacies) or (communit* adj2 setting*) or (healthcare adj2 setting*)).tw,kf,kw. 130394

6 ((Communit* adj1 hospital*) or (Public adj1 hospital*) or (Communit* adj1 clinic*) or (Walk-in adj1 clinic*) or (Public adj1 clinic*)).tw,kf,kw. 39766

7 Geriatrics/ 31350

8 Health services for the aged/ 18174

9 Geriatric assessment/ 32209

10 (Geriatric* or elder* or senior*).mp. 444715

11 or/1-10 720092

12 (Champion* or Cheerleader* or (peer* adj2 leader*) or (implementation adj2 coach*) or (implementation adj2 leader*) or (change adj2 leader*) or (change adj2 attribute*) or (change adj2 effort*)).mp. 13089

13 ((care adj2 coordinator*) or (care adj2 facilitator*) or (care adj2 aide*) or (care adj1 staff)).tw,kf,kw. 7293

14 or/12-13 20317

15 11 and 14 3051

16 limit 15 to dt=20130501-20230531 [Create date of May 1st, 2013 to May 31st, 2023] 1965

17 limit 15 to ed=20130501-20230531 [Entry date of May 1st, 2013 to May 31st, 2023] 1764

18 limit 15 to ep=20130501-20230531 [Electronic Date of Publication of May 1st, 2013 to May 31st, 2023] 1438

19 16 or 17 or 18 2025

## **OVID APA PsycINFO**

**Results Filtered to May 2013 onwards**

APA PsycInfo <1806 to May Week 4 2023>

1 Community Health/ or Community Services/ 22177

2 Community Mental Health Services/ 8134

3 Hospitals/ 17815

4 *Health Care Delivery/ 17368

5 ((communit* adj2 care) or (communit* adj2 intervention*) or (communit* adj2 treatment*) or (communit* adj2 health) or (communit* adj3 service*) or (communit* adj2 mental health*) or (communit* adj2 pharmacy) or (communit* adj2 pharmacies) or (communit* adj2 setting*) or (healthcare adj2 setting*)).tw. 63891

6 ((Communit* adj1 hospital*) or (Public adj1 hospital*) or (Communit* adj1 clinic*) or (Walk-in adj1 clinic*) or (Public adj1 clinic*)).tw. 6479

7 Geriatrics/ 12828

8 Geriatric Patients/ 14249

9 Geriatric Assessment/ 1178

10 (Geriatric* or elder* or senior*).mp. 143018

11 or/1-10 247405

12 (Champion* or Cheerleader* or (peer* adj2 leader*) or (implementation adj2 coach*) or (implementation adj2 leader*) or (change adj2 leader*) or (change adj2 attribute*) or (change adj2 effort*)).mp. 9327

13 ((care adj2 coordinator*) or (care adj2 facilitator*) or (care adj2 aide*) or (care adj1 staff)).tw. 3829

14 or/12-13 13133

15 11 and 14 1837

16 (201305* or 2014* or 2015* or 2016* or 2017* or 2018* or 2019* or 2020* or 2021* or 2022* or 2023*).dp,up. 1828656

17 15 and 16 932

## **OVID JBI EBP Database**

**Results Filtered to 2013 onwards**

JBI EBP Database <Current to May 17, 2023>

1 community health services/ 3

2 community mental health services/ or community pharmacy services/ 0

3 hospitals, community/ or hospitals, public/ 0

4 Delivery of Health Care/ 1

5 ((communit* adj2 care) or (communit* adj2 intervention*) or (communit* adj2 treatment*) or (communit* adj2 health) or (communit* adj3 service*) or (communit* adj2 mental health*) or (communit* adj2 pharmacy) or (communit* adj2 pharmacies) or (communit* adj2 setting*) or (healthcare adj2 setting*)).tw,kw. 1347

6 ((Communit* adj1 hospital*) or (Public adj1 hospital*) or (Communit* adj1 clinic*) or (Walk-in adj1 clinic*) or (Public adj1 clinic*)).tw,kw. 289

7 Geriatrics/ 3

8 Health services for the aged/ 0

9 Geriatric assessment/ 1

10 (Geriatric* or elder* or senior*).mp. 1489

11 or/1-10 2372

12 (Champion* or Cheerleader* or (peer* adj2 leader*) or (implementation adj2 coach*) or (implementation adj2 leader*) or (change adj2 leader*) or (change adj2 attribute*) or (change adj2 effort*)).mp. 98

13 ((care adj2 coordinator*) or (care adj2 facilitator*) or (care adj2 aide*) or (care adj1 staff)).tw,kw. 206

14 or/12-13 290

15 11 and 14 201

16 limit 15 to yr="2013 -Current" 145

## **EBSCO CINAHL Complete**

**Results Filtered to May 2013 onwards**

| **#** | **Query** | **Limiters/Expanders** | **Last Run Via** | **Results** |
| --- | --- | --- | --- | --- |
| S11 | S6 AND S9 | Limiters - Published Date: 20130501-20231231  Expanders - Apply equivalent subjects  Search modes - Boolean/Phrase | Interface - EBSCOhost Research Databases  Search Screen - Advanced Search  Database - CINAHL Complete | 4,490 |
| S10 | S6 AND S9 | Expanders - Apply equivalent subjects  Search modes - Boolean/Phrase | Interface - EBSCOhost Research Databases  Search Screen - Advanced Search  Database - CINAHL Complete | 7,381 |
| S9 | S7 OR S8 | Expanders - Apply equivalent subjects  Search modes - Boolean/Phrase | Interface - EBSCOhost Research Databases  Search Screen - Advanced Search  Database - CINAHL Complete | 76,717 |
| S8 | TX ((care N1 coordinator*) or (care N1 facilitator*) or (care N1 aide*) or (care N0 staff)) | Expanders - Apply equivalent subjects  Search modes - Boolean/Phrase | Interface - EBSCOhost Research Databases  Search Screen - Advanced Search  Database - CINAHL Complete | 28,000 |
| S7 | TX (Champion* or Cheerleader* or (peer* N1 leader*) or (implementation N1 coach*) or (implementation N1 leader*) or (change N1 leader*) or (change N1 attribute*) or (change N1 effort*)) | Expanders - Apply equivalent subjects  Search modes - Boolean/Phrase | Interface - EBSCOhost Research Databases  Search Screen - Advanced Search  Database - CINAHL Complete | 51,112 |
| S6 | S1 OR S2 OR S3 OR S4 OR S5 | Expanders - Apply equivalent subjects  Search modes - Boolean/Phrase | Interface - EBSCOhost Research Databases  Search Screen - Advanced Search  Database - CINAHL Complete | 352,810 |
| S5 | (TI Geriatric* or elder* or senior*) OR (AB Geriatric* or elder* or senior*) | Expanders - Apply equivalent subjects  Search modes - Boolean/Phrase | Interface - EBSCOhost Research Databases  Search Screen - Advanced Search  Database - CINAHL Complete | 175,553 |
| S4 | (MH "Geriatrics") OR (MH "Health Services for Older Persons") OR (MH "Geriatric Assessment") | Expanders - Apply equivalent subjects  Search modes - Boolean/Phrase | Interface - EBSCOhost Research Databases  Search Screen - Advanced Search  Database - CINAHL Complete | 25,444 |
| S3 | TI ( ((Communit* N0 hospital*) or (Public N0 hospital*) or (Communit* N0 clinic*) or (Walk-in N0 clinic*) or (Public N0 clinic*)) ) OR AB ( ((Communit* N0 hospital*) or (Public N0 hospital*) or (Communit* N0 clinic*) or (Walk-in N0 clinic*) or (Public N0 clinic*)) ) | Expanders - Apply equivalent subjects  Search modes - Boolean/Phrase | Interface - EBSCOhost Research Databases  Search Screen - Advanced Search  Database - CINAHL Complete | 18,718 |
| S2 | TI ( ((communit* N1 care) or (communit* N1 intervention*) or (communit* N1 treatment*) or (communit* N1 health) or (communit* N2 service*) or (communit* N1 mental health*) or (communit* N1 pharmacy) or (communit* N1 pharmacies) or (communit* N1 setting*) or (healthcare N1 setting*)) ) OR AB ( ((communit* N1 care) or (communit* N1 intervention*) or (communit* N1 treatment*) or (communit* N1 health) or (communit* N2 service*) or (communit* N1 mental health*) or (communit* N1 pharmacy) or (communit* N1 pharmacies) or (communit* N1 setting*) or (healthcare N1 setting*)) ) | Expanders - Apply equivalent subjects  Search modes - Boolean/Phrase | Interface - EBSCOhost Research Databases  Search Screen - Advanced Search  Database - CINAHL Complete | 84,549 |
| S1 | (MH "Community Health Services") OR (MH "Community Mental Health Services") OR (MH "Community Health Centers") OR (MH "Hospitals, Community") OR (MH "Hospitals, Public") OR (MM "Health Care Delivery") | Expanders - Apply equivalent subjects  Search modes - Boolean/Phrase | Interface - EBSCOhost Research Databases  Search Screen - Advanced Search  Database - CINAHL Complete | 95,695 |

## **Wiley’s Cochrane Library**

**Results Filtered to May 2013 onwards**

ID Search Hits

#1 MeSH descriptor: [Community Health Services] this term only 1253

#2 MeSH descriptor: [Community Mental Health Services] this term only 846

#3 MeSH descriptor: [Community Pharmacy Services] this term only 333

#4 MeSH descriptor: [Hospitals, Community] this term only 189

#5 MeSH descriptor: [Hospitals, Public] this term only 192

#6 MeSH descriptor: [Delivery of Health Care] this term only 1343

#7 (((communit* Near/2 care) or (communit* Near/2 intervention*) or (communit* Near/2 treatment*) or (communit* Near/2 health) or (communit* Near/3 service*) or (communit* Near/2 mental health*) or (communit* Near/2 pharmacy) or (communit* Near/2 pharmacies) or (communit* Near/2 setting*) or (healthcare Near/2 setting*))):ti,ab,kw 20742

#8 (((Communit* Near/1 hospital*) or (Public Near/1 hospital*) or (Communit* Near/1 clinic*) or (Walk-in Near/1 clinic*) or (Public Near/1 clinic*))):ti,ab,kw 4324

#9 MeSH descriptor: [Geriatrics] this term only 398

#10 MeSH descriptor: [Health Services for the Aged] this term only 537

#11 MeSH descriptor: [Geriatric Assessment] this term only 1888

#12 (Geriatric* or elder* or senior*) 78876

#13 #1 OR #2 OR #3 OR #4 OR #5 OR #6 OR #7 OR #8 OR #9 OR #10 OR #11 OR #12 101869

#14 (Champion* or Cheerleader* or (peer* Near/2 leader*) or (implementation Near/2 coach*) or (implementation Near/2 leader*) or (change Near/2 leader*) or (change Near/2 attribute*) or (change Near/2 effort*)) 1828

#15 (((care Near/2 coordinator*) or (care Near/2 facilitator*) or (care Near/2 aide*) or (care Near/1 staff))):ti,ab,kw 1033

#16 #14 OR #15 2841

#17 #13 AND #16 with Cochrane Library publication date Between May 2013 and Dec 2023 574

**[Reviews: 56]**

**[Trials: 515]**

**[Protocols: 2]**

**[Clinical Answers: 1]**

## **NLM’s PubMed**

**Results Filtered to 2013 onwards**

((((((((((community health services[MeSH Terms]) OR (community mental health services[MeSH Terms])) OR (community pharmacy services[MeSH Terms])) OR (hospitals, community[MeSH Terms])) OR (hospitals, public[MeSH Terms])) OR (Delivery of Health Care[MeSH Major Topic])) OR (("community care"[Title/Abstract:~2] or "community interventions"[Title/Abstract:~2] or "community intervention"[Title/Abstract:~2] or "community treatment"[Title/Abstract:~2] or "community treatments"[Title/Abstract:~2] or "community health"[Title/Abstract:~2] or "community services"[Title/Abstract:~3] or "community mental health"[Title/Abstract:~2] or "community pharmacy"[Title/Abstract:~2] or "community pharmacies"[Title/Abstract:~2] or "community settings"[Title/Abstract:~2] or "healthcare settings"[Title/Abstract:~2]))) OR (("community hospitals"[Title/Abstract:~2] or "public hospitals"[Title/Abstract:~2] or "community clinics"[Title/Abstract:~2] or "walk-in clinic"[Title/Abstract:~2] or "public clinic"[Title/Abstract:~2]))) OR (((Geriatrics[MeSH Terms]) OR (health services for the aged[MeSH Terms])) OR (geriatric assessment[MeSH Terms]))) OR (Geriatric* or elder* or senior*)) AND (((Champion* or Cheerleader*) OR (("peer leaders"[Title/Abstract:~2] or "implementation coach"[Title/Abstract:~2] or "implementation leaders"[Title/Abstract:~2] or "change leaders"[Title/Abstract:~2] or "change attributes"[Title/Abstract:~2] or "change efforts"[Title/Abstract:~2]))) OR (("care coordinator"[Title/Abstract:~2] or "care facilitator"[Title/Abstract:~2] or "care aides"[Title/Abstract:~2] or "care staff"[Title/Abstract:~2]))) AND (pubstatusaheadofprint OR publisher[SB] or in process[SB] or pubmednotmedline[SB])

Filters: **from 2013 - 2023**

**Results: 466; Searched on May 31, 2023 @ 3:53PM EST**

## **Elsevier’s Scopus**

**Results Filtered to 2013 onwards**

( ( TITLE-ABS-KEY ( champion* OR cheerleader* ) ) OR ( TITLE-ABS-KEY ( ( peer* W/1 leader* ) OR ( implementation W/1 coach* ) OR ( implementation W/1 leader* ) OR ( change W/1 leader* ) OR ( change W/1 attribute* ) OR ( change W/1 effort* ) OR ( care W/1 coordinator* ) OR ( care W/1 facilitator* ) OR ( care W/1 aide* ) OR ( care W/0 staff ) ) ) ) AND ( ( TITLE-ABS-KEY ( delivery AND of AND healthcare ) ) OR ( TITLE-ABS-KEY ( ( communit* W/1 care ) OR ( communit* W/1 intervention* ) OR ( communit* W/1 treatment* ) OR ( communit* W/1 health ) OR ( communit* W/2 service* ) OR ( communit* W/1 mental AND health* ) OR ( communit* W/1 pharmacy ) OR ( communit* W/1 pharmacies ) OR ( communit* W/1 setting* ) OR ( healthcare W/1 setting* ) ) ) OR ( TITLE-ABS-KEY ( ( ( communit* W/0 hospital* ) OR ( public W/0 hospital* ) OR ( communit* W/0 clinic* ) OR ( walk-in W/0 clinic* ) OR ( public W/0 clinic* ) ) ) ) OR ( TITLE-ABS-KEY ( health AND services AND for AND the AND aged ) ) OR ( TITLE-ABS-KEY ( geriatric* OR elder* OR senior* ) ) ) AND ( LIMIT-TO ( PUBYEAR , 2023 ) OR LIMIT-TO ( PUBYEAR , 2022 ) OR LIMIT-TO ( PUBYEAR , 2021 ) OR LIMIT-TO ( PUBYEAR , 2020 ) OR LIMIT-TO ( PUBYEAR , 2019 ) OR LIMIT-TO ( PUBYEAR , 2018 ) OR LIMIT-TO ( PUBYEAR , 2017 ) OR LIMIT-TO ( PUBYEAR , 2016 ) OR LIMIT-TO ( PUBYEAR , 2015 ) OR LIMIT-TO ( PUBYEAR , 2014 ) OR LIMIT-TO ( PUBYEAR , 2013 ) )

**Searched on May 31, 2023 @3:08PM EST. Results: 3958**

## **Clarivate’s Web of Science Core Collection:**

**Results Filtered to 2013 onwards**

# Web of Science Search Strategy (v0.1)

# Database: Web of Science Core Collection

# Entitlements:

- WOS.SCI: 1900 to 2023

- WOS.AHCI: 1975 to 2023

- WOS.BHCI: 2005 to 2023

- WOS.BSCI: 2005 to 2023

- WOS.ESCI: 2005 to 2023

- WOS.ISTP: 1990 to 2023

- WOS.SSCI: 1900 to 2023

- WOS.ISSHP: 1990 to 2023

# Searches:

1: (((((TS=(community health services)) OR TS=(community mental health services)) OR TS=(hospitals, community)) OR TS=(community pharmacy services)) OR TS=(hospitals, public)) OR TS=(Delivery of Health Care) Date Run: Wed May 31 2023 14:43:21 GMT-0400 (Eastern Daylight Time) Results: 293598

2: TS=(((communit* Near/1 care) or (communit* Near/1 intervention*) or (communit* Near/1 treatment*) or (communit* Near/1 health) or (communit* Near/2 service*) or (communit* Near/1 mental health*) or (communit* Near/1 pharmacy) or (communit* Near/1 pharmacies) or (communit* Near/1 setting*) or (healthcare Near/1 setting*))) Date Run: Wed May 31 2023 14:44:50 GMT-0400 (Eastern Daylight Time) Results: 145946

3: TS=(((Communit* Near/0 hospital*) or (Public Near/0 hospital*) or (Communit* Near/0 clinic*) or (Walk-in Near/0 clinic*) or (Public Near/0 clinic*))) Date Run: Wed May 31 2023 14:45:32 GMT-0400 (Eastern Daylight Time) Results: 38776

4: ((TS=(Geriatrics)) OR TS=(Health services for the aged)) OR TS=(Geriatric assessment) Date Run: Wed May 31 2023 14:45:53 GMT-0400 (Eastern Daylight Time) Results: 116898

5: TS=((Geriatric* or elder* or senior*)) Date Run: Wed May 31 2023 14:46:06 GMT-0400 (Eastern Daylight Time) Results: 537260

6: #5 OR #4 OR #3 OR #2 OR #1 Date Run: Wed May 31 2023 14:46:12 GMT-0400 (Eastern Daylight Time) Results: 959712

7: ALL=((Champion* or Cheerleader* or (peer* "Near/1" leader*) or (implementation "Near/1" coach*) or (implementation "Near/1" leader*) or (change "Near/1" leader*) or (change "Near/1" attribute*) or (change "Near/1" effort*))) Date Run: Wed May 31 2023 14:47:13 GMT-0400 (Eastern Daylight Time) Results: 38449

8: ALL=(((care "Near/1" coordinator*) or (care "Near/1" facilitator*) or (care "Near/1" aide*) or (care "Near/0" staff))) Date Run: Wed May 31 2023 14:47:43 GMT-0400 (Eastern Daylight Time) Results: 4

9: #8 OR #7 Date Run: Wed May 31 2023 14:47:49 GMT-0400 (Eastern Daylight Time) Results: 38451

10: #9 AND #6 Date Run: Wed May 31 2023 14:48:05 GMT-0400 (Eastern Daylight Time) Results: 1913

11: #9 AND #6 and 2023 or 2022 or 2021 or 2020 or 2019 or 2018 or 2017 or 2016 or 2015 or 2014 or 2013  (Publication Years) Date Run: Wed May 31 2023 14:48:33 GMT-0400 (Eastern Daylight Time) Results: 1454
