# Supplementary material for: Exploring the roles and functions of champions within community-based interventions to support older adults with chronic conditions: A scoping review protocol
Source: PLoS One. 2023 Oct 13;18(10):e0291252. doi: 10.1371/journal.pone.0291252 (PMC10575514; doi:10.1371/journal.pone.0291252)
Supplement: S1 Appendix — (DOCX) [file pone.0291252.s002.docx]

**Appendix A: Ovid MEDLINE(R) ALL**

1              aged/ or "aged, 80 and over"/ or centenarians/ or nonagenarians/ or octogenarians/ or frail elderly/                3437386

2              Health services for the aged/ or Geriatric assessment/   48504

3              ((Old adj2 Adult*) or (Old adj2 Person*) or (Old adj2 People*) or (Old adj2 Patient*) or (Old adj2 Citizen*) or (Older adj2 Adult*) or (Older adj2 Person*) or (Older adj2 People*) or (Older adj2 Patient*) or (Older adj2 Citizen*) or (Oldest adj2 Adult*) or (Oldest adj2 Person*) or (Oldest adj2 People*) or (Oldest adj2 Patient*) or (Oldest adj2 Citizen*) or (Ag?ing adj2 Adult*) or (Ag?ing adj2 Person*) or (Ag?ing adj2 People*) or (Ag?ing adj2 Patient*) or (Ag?ing adj2 Citizen*) or (Aged adj2 Adult*) or (Aged adj2 Person*) or (Aged adj2 People*) or (Aged adj2 Patient*) or (Aged adj2 Citizen*)).tw,kf,kw. 476290

4              (Oldest Old or Elder* or Geriatric* or Senior* or Long-Lived or Over-the-hill or Senescen* or Centenarian or Nonagenarian or Octogenarian or Septuagenarian or Sexagenerian or Unyoung).tw,kf,kw.          475562

5              ((Aged adj "60") or (Aged adj "70") or (Aged adj "75") or (Aged adj "80") or (Aged adj "85") or (Aged adj "90") or (Aged adj "95") or (Aged adj "100") or (Aged adj sixty) or (Aged adj sixty-five) or (Aged adj seventy) or (Aged adj seventy-five) or (Aged adj eighty) or (Aged adj eighty-five) or (Aged adj ninety) or (Aged adj ninety-five) or (Aged adj one hundred) or (Aged adj a hundred)).tw,kf,kw.    42969

6              1 or 2 or 3 or 4 or 5           3862779

7 hospitals, community/ or hospitals, public/          29180

8          ((Communit* adj1 hospital*) or (Public adj1 hospital*) or (Communit* adj1 clinic*) or (Walk-in adj1 clinic*) or (Public adj1 clinic*)).tw,kf,kw.   39080

9          7 or 8 145498

10           Community Health Services/      32994

11           Champion*.tw,kf,kw.    9413

12           ((Community* adj2 care) or (community adj2 intervention*)).tw,kf,kw.                24618

13           ((care adj2 coordinator*) or (care adj2 facilitator*) or (care adj2 aide*) or (care adj1 staff)).tw,kf,kw.      7139

14           10 or 11 or 12 or 13          70694

15           6 and 9 and 14 2768

16           limit 18 to yr="2013 -Current"     1293
